# Structural volumetric and Periodic Table DTI patterns in Complex Normal Pressure Hydrocephalus—Toward the principles of a translational taxonomy

**DOI:** 10.3389/fnhum.2024.1188533

**Published:** 2024-03-13

**Authors:** Christine Lock, Emma M. S. Toh, Nicole C. Keong

**Affiliations:** ^1^Department of Neurosurgery, National Neuroscience Institute, Singapore, Singapore; ^2^Yong Loo Lin School of Medicine, National University of Singapore, Singapore, Singapore; ^3^Duke-NUS Medical School, Singapore, Singapore

**Keywords:** normal pressure hydrocephalus (NPH), magnetic resonance imaging (MRI), diffusion tensor imaging (DTI), white matter, injury properties

## Abstract

**Introduction:**

We previously proposed a novel taxonomic framework to describe the diffusion tensor imaging (DTI) profiles of white matter tracts by their diffusivity and neural properties. We have shown the relevance of this strategy toward interpreting brain tissue signatures in Classic Normal Pressure Hydrocephalus vs. comparator cohorts of mild traumatic brain injury and Alzheimer’s disease. In this iteration of the Periodic Table of DTI Elements, we examined patterns of tissue distortion in Complex NPH (*Co*NPH) and validated the methodology against an open-access dataset of healthy subjects, to expand its accessibility to a larger community.

**Methods:**

DTI measures for 12 patients with *Co*NPH with multiple comorbidities and 45 cognitively normal controls from the ADNI database were derived using the image processing pipeline on the brainlife.io open cloud computing platform. Using the Periodic Table algorithm, DTI profiles for *Co*NPH vs. controls were mapped according to injury patterns.

**Results:**

Structural volumes in most structures tested were significantly lower and the lateral ventricles higher in *Co*NPH vs. controls. In *Co*NPH, significantly lower fractional anisotropy (FA) and higher mean, axial, and radial diffusivities (MD, L1, and L2 and 3, respectively) were observed in white matter related to the lateral ventricles. Most diffusivity measures across supratentorial and infratentorial structures were significantly higher in *Co*NPH, with the largest differences in the cerebellum cortex. In subcortical deep gray matter structures, *Co*NPH and controls differed most significantly in the hippocampus, with the *Co*NPH group having a significantly lower FA and higher MD, L1, and L2 and 3. Cerebral and cerebellar white matter demonstrated more potential reversibility of injury compared to cerebral and cerebellar cortices.

**Discussion:**

The findings of widespread and significant reductions in subcortical deep gray matter structures, in comparison to healthy controls, support the hypothesis that Complex NPH cohorts retain imaging features associated with Classic NPH. The use of the algorithm of the Periodic Table allowed for greater consistency in the interpretation of DTI results by focusing on patterns of injury rather than an over-reliance on the interrogation of individual measures by statistical significance alone. Our aim is to provide a prototype that could be refined for an approach toward the concept of a “translational taxonomy.”

## 1 Introduction

The capacity of brain matter to recover from injury via neural regeneration and natural mechanisms of repair is finite. Augmenting this recovery by artificial introduction of novel therapies, such as drug interventions and brain implants, is a subject of intense research endeavor. Yet, the excitement in such efforts masks a flawed underlying assumption; that all brain injury has equivalent potential to respond to effective interventions.

An understanding of gross structural features of brain injury, along with knowledge of disease trajectories of neuropathological processes, may be insufficient to address this area of concern. It should instead be appreciated that brain injury exists along a spectrum of likely reversibility to irreversibility. The degree to which this spectrum is expressed within a disease process may vary. This variation of expression may also be dependent upon temporal factors, such as whether the onset of injury was acute vs. chronic, and the rapidity of clinical stages that may occur in between.

Some patterns of neurological disease are more reversible than others, for example, compression of white matter tracts by brain tumors may be more amenable to surgical intervention than their destruction by malignant infiltration or radiotherapy-induced degeneration. One neurological condition that exhibits the breadth of brain injury patterns from acute to chronic, and reversible to irreversible, is hydrocephalus, in which excessive water accumulates in the brain from a disturbance affecting the circulation of cerebrospinal fluid (CSF). In normal pressure hydrocephalus (NPH), the process leading to the accumulation of brain fluid may be slow, and so, the accumulation of damages from brain injury may be insidious. Yet, the injury is reversible. Patients with NPH classically present with a triad of symptoms (often asynchronous or incomplete) comprising gait and balance disturbance, cognitive dysfunction and urinary incontinence, that improves with CSF diversion in the form of a surgically implanted shunt device.

The pathophysiology of NPH and the reason for its reversibility are still debated; NPH also appears to exist along a spectrum of disease. Whilst idiopathic NPH (iNPH), the diagnosis of which must meet published criteria according to the NPH guidelines (Relkin et al., 2005; Nakajima et al., 2021), serves as a human model of reversible brain injury, other subtypes of presentation exist. This includes NPH with significant overlay from comorbidities (Malm et al., 2013) such as neurodegenerative disorders and vascular risks, and may be more clinically challenging to unravel. To distinguish these cohorts, we have previously proposed the terminology of Classic vs. Complex NPH (*Cl*NPH vs. *Co*NPH, respectively; Lock et al., 2019; Goh et al., 2022). In *Co*NPH, concurrent risk factors for neurological decline may complicate and confound the assessment of CSF responsiveness. Here, the use of diffusion tensor imaging (DTI) has allowed for an unprecedented level of understanding about the multiple patterns of brain injury co-existing in a single presentation of disease. DTI interrogates the microstructural architecture of brain tissue and in particular, white matter patterns of injury. In NPH, we and other groups have demonstrated such patterns of injury may suggest mechanisms such as compression, stretch, edema and changes compatible with neurodegeneration. There is potential for DTI to contribute toward transforming our approach to the assessment of the capacity of patients to respond to interventions and to document the effectiveness of those therapies.

A major hurdle to the more widespread use of DTI as such a tool is its perceived lack of transparency and reproducibility of results. DTI measures derived for analysis may not be comparable between sites or across groups. DTI output is highly dependent upon technical factors, such as machine-specific values for scanning acquisition and software used for post-processing. To address this problem and harness the full potential of DTI in NPH, solutions must be found to ensure DTI measures can be used reliably on their own or meaningfully correlated to other more established imaging markers. In our previous work, we were the first group to construct a strategy which we termed a Periodic Table of DTI Elements. Toward this novel language of interpretation, we organized DTI profiles reported by our group and others into a common taxonomic framework which described white matter tracts by their diffusivity and neural properties. We then confirmed its relevance to cohorts of Classic NPH (*Cl*NPH) vs. comparator cohorts of mild traumatic brain injury (TBI) and Alzheimer’s disease (AD) (Keong et al., 2022).

In this study, we sought to examine its utility in Complex NPH (*Co*NPH) and validate the methodology of the Periodic Table of DTI Elements against an open-access dataset of healthy control subjects to promote its potential accessibility to a wider community of users. We aimed to—(i) examine patterns of tissue distortion in *Co*NPH vs. healthy controls by comparing differences in brain volumes and DTI profiles, (ii) expand the application of the Periodic Table of DTI Elements to include other cortical and subcortical regions-of-interest (ROIs) and (iii) provide baseline values in comparison to healthy controls derived from the ADNI project.

## 2 Materials and methods

Thirteen patients with Complex NPH who were scheduled to undergo an extended CSF drainage protocol at the National Neuroscience Institute were recruited in 2016 and 2017. These patients had clinical symptoms consistent with probable/possible NPH according to international guidelines (Marmarou et al., 2005; Relkin et al., 2005), had neurological features supporting the NPH diagnosis, and presented with multiple comorbidities, including overlay from neurodegenerative diseases and vascular risk burden. Pre-drainage, patients had baseline Magnetic Resonance Imaging (MRI) and clinical gait and cognitive testing. One patient was excluded from the analysis as they were unable to complete MR imaging. The NPH extended CSF drainage protocol and clinical characteristics of this group of *Co*NPH patients have been described in a previous paper (Lock et al., 2019).

MR images for cognitively normal controls were downloaded from the Alzheimer’s Disease Neuroimaging Initiative (ADNI) database^1^ in January 2020. Subjects who had baseline MRI scans with DTI sequences from the ADNI 1, ADNI GO, and ADNI 2 study phases were selected. DTI measures were derived for the NPH and control scans using the image processing pipeline on the cloud-computing platform brainlife.io. DTI profiles based on differences between cohorts were then mapped according to their injury patterns, using the hierarchical algorithm of the Periodic Table of DTI Elements. In this study, we refined the algorithm published in our previous work to include a descriptor of morphological DTI profile heretofore unseen prior to the comparison of cohorts presented here.

### 2.1 MRI acquisition

MR imaging data for NPH subjects were acquired with a 3.0-T MR scanner (Ingenia, Philips Medical Systems, Best, Netherlands), including 3D T1, T2, FLAIR, and DTI sequences. DTI was obtained using a single-shot echo-planar sequence with a slice thickness of 2.3 mm. Images were acquired in 20 gradient directions with the following parameters: *b* = 0 and 1,000 s/mm^2^, TR = 7,274 ms; TE = 80 ms; FOV 220 mm × 220 mm; and matrix = 96 × 96, resulting in a voxel size of 2.3 mm × 2.3 mm × 2.3 mm, with SENSE factor of 2.5. A few patients were downgraded to the 1.5-T scanner at equivalent specifications due to MR safety concerns. Cognitively normal controls from the ADNI study were scanned on 3T GE Medical Systems scanners at 14 acquisition sites in North America (Nir et al., 2013). Diffusion weighted images were acquired with the following parameters: 256 × 256 matrix; voxel size: 2.7 mm × 2.7 mm × 2.7 mm; TR = 9,000 ms; scan time = 9 min. Forty-six separate images were acquired for each DTI scan: 5 T2-weighted images with no diffusion sensitization (b0 images) and 41 diffusion-weighted images (*b* = 1,000 s/mm^2^).

### 2.2 Pre- and post-processing of anatomical (T1w) and diffusion (dMRI) imaging datasets

Local NPH imaging datasets were anonymized at source, then subject to further deidentification, defacing and processing prior to usage. Using the fsl_anat functionality (brainlife.app.273) of the FMRIB Software Library (FSL) (Smith et al., 2004; Woolrich et al., 2009; Jenkinson et al., 2012), anatomical preprocessing of the deidentified T1-weighted (T1w) images was performed. The anatomical T1w images were cropped and reoriented to match the MNI152 template, then subjected to linear and non-linear alignment using the FLIRT tool (Jenkinson and Smith, 2001; Jenkinson et al., 2002; Greve and Fischl, 2009). The linearly aligned images were used as the acpc aligned T1w images for further post-processing with Freesurfer (Dale and Sereno, 1993; Sled et al., 1998; Dale et al., 1999; Fischl et al., 1999a,b, 2001, 2002, 2004a,b; Fischl and Dale, 2000; Rosas et al., 2002; Kuperberg et al., 2003; Salat et al., 2004; Segonne et al., 2004, 2007; Desikan et al., 2006; Han et al., 2006; Jovicich et al., 2006; Reuter et al., 2010, 2012; Reuter and Fischl, 2011). In this study, we preferred Freesurfer 7.1.1 (brainlife.app.462) over 7.3.2 as we found that the earlier release version generated fewer segmentation failures in our NPH dataset. Accordingly, ADNI datasets were processed using the same release. The recon_all function was used to generate pial/cortical and white matter surfaces and perform brain parcellations according to known neuroanatomical atlases. The Destrieux (aparc.a20090s) atlas was used for subsequent segmentation of white matter and mapping of diffusion metrics to the relevant cortical and subcortical structures.

Supplementary processing of the deidentified images was performed using processing pipelines via the brainlife.io secure cloud processing platform, as per methodology described by Caron et al. (2021). In brief, following manual inspection of the images, additional FSL and Freesurfer steps via their equivalent brainlife apps were repeated as necessary to achieve satisfactory brain segmentation. Preprocessing of dMRI data was performed using MRTrix3, with alignment of the dMRI images to the acpc aligned T1w dataset (brainlife.app.68). MRTrix3—Anatomically constrained probabilistic tractography (ACT) (brainlife.app.319) was used to generate white matter tractography (Smith et al., 2012; Takemura et al., 2016; Ades-Aron et al., 2018; Avesani et al., 2019; Tournier et al., 2019). Following this, cortex tissue mapping (brainlife.app.381) was performed as per Fukutomi et al. (2018) using dwi, freesurfer and tensor output from the processing steps above. Summary measures for the processing pipeline were then generated using the Freesurfer Statistics (brainlife.app.272) and compute summary statistics of diffusion measures from subcortical (brainlife.app.389) and cortical segmentation (brainlife.app.483) brainlife apps for further analyses (Dale et al., 1999; Fukutomi et al., 2018; Avesani et al., 2019). At each stage of the pipeline, images were visually inspected and if necessary, manually reprocessed to optimize output. The methodology is described in more detail in Lock et al. (2024), and the open-source code for each app used is linked in Table 1.

**TABLE 1:** Applications on brainlife.io used for dMRI processing.

Application	Link to online app
Anatomical alignment	https://doi.org/10.25663/brainlife.app.273
Brainlife wrapper for Freesurfer	https://doi.org/10.25663/brainlife.app.462
Freesurfer statistics	https://doi.org/10.25663/brainlife.app.272
dMRI preprocessing	https://doi.org/10.25663/bl.app.68
MRTrix3	https://doi.org/10.25663/brainlife.app.319
Cortex tissue mapping	https://doi.org/10.25663/brainlife.app.381
Compute subcortical diffusion measures	https://doi.org/10.25663/brainlife.app.389
Compute cortical diffusion measures	https://doi.org/10.25663/brainlife.app.483

### 2.3 Statistical analysis

Statistical analyses were conducted using SPSS Statistics Version 23.0 (IBMCorp., Armonk, NY, USA). Data is reported as mean ± standard deviation, unless otherwise stated. DTI metrics and volumes were compared between the *Co*NPH and cognitively normal control groups using the Mann-Whitney *U*-test and corrected for multiple comparisons with a false discovery rate (FDR) of 0.05. *P*-values reported are unadjusted; after controlling for FDR, p is significant at ≤0.033.

### 2.4 Graphical visualization of DTI profiles

A novel visualization method was used to better illustrate the white matter injury patterns across selected orders of ROIs. The Mayavi package (v4.8.0) with Python (v3.9.13) was used to generate 3-Dimensional visualizations. Each visualization consisted of three aspects and incorporated DTI metrics of fractional anisotropy (FA), mean diffusivity (MD), axial diffusivity (L1), and radial diffusivity (L2 and 3). For each ROI, the visualizations were averaged across *Co*NPH patients and cognitively normal controls. The first aspect, i.e., the outer layer, is in the shape of a ellipsoid with discontinuity and is generated using all diffusivity measures (FA, MD, L1 and L2 and 3). Indeed, the resulting shape may be considered a simplistic graphical representation of a tensor ellipse, as its directionality represents FA. The second aspect is the texture, i.e., porosity, of the spherical structure, represented by non-FA diffusivities. The more porous the texture of the sphere, the greater the disruption in tissue or structural integrity, mainly reflecting the axial and radial diffusivity measures. The third aspect, the size of the sphere, represents the degree of diffusivity in both directional (L1 and L2 and 3) and global (MD) diffusivities. The larger the amplitude of MD, L1 and/or L2 and 3, the larger the size of the sphere.

## 3 Results

The *Co*NPH patient group consisted of 12 patients (10 male and 2 female; mean age 71.3 ± 7.57 years). A total of 45 cognitively normal controls from the ADNI study were included in this analysis (21 male and 24 female; mean age 72.8 ± 6.09 years). The *Co*NPH and cognitively normal groups were not significantly different in age.

### 3.1 Structural volumes

Mean structural volumes for NPH patients and controls are shown in Table 2. In the lateral ventricles, structural volumes were significantly higher in *Co*NPH and more than 1.5 times of that compared to controls (*p* < 0.001 for right and left lateral ventricles and inferior lateral ventricles). Volumes of most other structures tested were significantly lower in *Co*NPH compared to healthy controls, except for the right and left caudate, right thalamus, right putamen, and left accumbens (*p* < 0.001 for right and left cerebral white matter and cortex; *p* ≤ 0.004 for right and left cerebellum white matter and cortex; *p* ≤ 0.001 for corpus callosum; *p* = 0.007 for left thalamus; *p* = 0.030 for left putamen; *p* = 0.004 for right hippocampus and *p* = 0.028 for left hippocampus).

**TABLE 2:** Structural volumes (voxels) in NPH patients and controls.

Structural volumes	Controls	NPH	*p*
Lateral ventricle (R)	14464 ± 6001	45092 ± 18586	**<0**.**001**
Lateral ventricle (L)	15103 ± 5833	43543 ± 19098	**<0**.**001**
Inferior lateral ventricle (R)	598 ± 394	2780 ± 1463	**<0**.**001**
Inferior lateral ventricle (L)	581 ± 343	2650 ± 1146	**<0**.**001**
Cerebral white matter (R)	205473 ± 22312	177144 ± 17363	**<0**.**001**
Cerebral white matter (L)	204904 ± 21839	173683 ± 19835	**<0**.**001**
Cerebral cortex (R)	221639 ± 18964	189324 ± 16106	**<0**.**001**
Cerebral cortex (L)	221929 ± 18472	190763 ± 18862	**<0**.**001**
Cerebellum white matter (R)	10826 ± 2096	8720 ± 1704	**0**.**004**
Cerebellum white matter (L)	12230 ± 7575	9110 ± 1562	**0**.**002**
Cerebellum cortex (R)	52120 ± 5272	46121 ± 4248	**0**.**001**
Cerebellum cortex (L)	51745 ± 5048	45461 ± 4117	**<0**.**001**
Anterior corpus callosum	905 ± 153	595 ± 264	**<0**.**001**
Central corpus callosum	475 ± 79.1	343 ± 108	**0**.**001**
Posterior corpus callosum	1019 ± 138	624 ± 372	**<0**.**001**
Caudate (R)	3354 ± 598	3431 ± 1418	0.667
Caudate (L)	3219 ± 452	3210 ± 1296	0.422
Thalamus (R)	6076 ± 702	5601 ± 1067	0.092
Thalamus (L)	6471 ± 793	5227 ± 1543	**0**.**007**
Putamen (R)	4304 ± 540	4009 ± 977	0.078
Putamen (L)	4159 ± 586	3718 ± 719	**0**.**030**
Hippocampus (R)	3918 ± 423	3482 ± 427	**0**.**004**
Hippocampus (L)	3832 ± 418	3270 ± 898	**0**.**028**

Data reported as Mean ± SD; (R) for right side and (L) for left side. *P*-values in bold are significant.

### 3.2 Supratentorial and infratentorial structures

Diffusion tensor imaging metrics (FA, MD, L1, L2 and 3) across right and left sided supratentorial and infratentorial structures were significantly higher in *Co*NPH compared to controls, with the exception of FA in the right and left cerebral cortex and L2 and 3 in the left cerebral white matter (Table 3). The largest differences were in the cerebellum cortex, where FA, MD, L1, and L2 and 3 in *Co*NPH were 2 times that of controls (*p* < 0.001 for all).

**TABLE 3:** Diffusion tensor imaging (DTI) metrics for white matter vs. cortical matter in the supratentorial vs. infratentorial spaces in NPH patients and controls.

Structure	Controls	NPH	% difference*	*p*
**Cerebral white matter (R)**				
FA	0.205 ± 0.074	0.299 ± 0.057	[ + 45.9]	**0**.**001**
MD	7.77 ± 1.43	9.71 ± 1.52	+ 25.1	**0**.**001**
L1	9.83 ± 1.83	12.77 ± 1.31	+ 30.0	**<0**.**001**
L2 and 3	6.74 ± 1.30	8.18 ± 1.63	+ 21.5	**0**.**007**
**Cerebral white matter (L)**				
FA	0.205 ± 0.074	0.307 ± 0.059	[ + 49.2]	**0**.**002**
MD	8.07 ± 1.50	9.38 ± 0.910	+ 16.2	**0**.**005**
L1	10.19 ± 1.88	12.47 ± 0.610	+ 22.5	**<0**.**001**
L2 and 3	7.02 ± 1.37	7.84 ± 1.08	+ 11.7	0.081
**Cerebral cortex (R)**				
FA	0.143 ± 0.023	0.154 ± 0.019	+ 7.94	0.248
MD	7.38 ± 2.04	11.59 ± 0.766	+ 57.1	**<0**.**001**
L1	8.88 ± 2.23	13.29 ± 0.781	+ 49.6	**<0**.**001**
L2 and 3	6.63 ± 1.94	10.75 ± 0.778	[ + 62.0]	**<0**.**001**
**Cerebral cortex (L)**				
FA	0.141 ± 0.030	0.159 ± 0.026	+13.2	0.096
MD	7.72 ± 1.93	11.54 ± 0.813	+49.5	**<0**.**001**
L1	9.22 ± 2.08	13.29 ± 0.757	+ 44.2	**<0**.**001**
L2 and 3	6.74 ± 2.09	10.66 ± 0.861	[ + 58.1]	**<0**.**001**
**Cerebellum white matter (R)**				
FA	0.141 ± 0.133	0.380 ± 0.048	[ + 170.0]	**<0**.**001**
MD	5.12 ± 3.39	9.71 ± 3.83	+ 89.6	**<0**.**001**
L1	6.47 ± 4.44	13.63 ± 4.43	+ 110.5	**<0**.**001**
L2 and 3	4.44 ± 2.91	7.75 ± 3.53	+ 74.4	**0**.**007**
**Cerebellum white matter (L)**				
FA	0.133 ± 0.130	0.383 ± 0.052	[ + 189.0]	**<0**.**001**
MD	5.18 ± 3.46	9.74 ± 3.81	+ 88.2	**<0**.**001**
L1	6.49 ± 4.48	13.60 ± 4.01	+ 109.5	**<0**.**001**
L2 and 3	4.52 ± 2.99	7.82 ± 3.72	+ 73.0	**0**.**020**
**Cerebellum cortex (R)**				
FA	0.065 ± 0.064	0.188 ± 0.020	+ 190.4	**<0**.**001**
MD	3.28 ± 3.31	11.01 ± 1.24	+ 235.3	**<0**.**001**
L1	3.93 ± 3.91	13.04 ± 1.54	+ 232.1	**<0**.**001**
L2 and 3	2.96 ± 3.01	10.00 ± 1.11	[ + 237.5]	**<0**.**001**
**Cerebellum cortex (L)**				
FA	0.065 ± 0.062	0.185 ± 0.017	+ 184.9	**<0**.**001**
MD	3.38 ± 3.24	11.28 ± 1.50	+ 234.1	**<0**.**001**
L1	4.03 ± 3.83	13.28 ± 1.72	+ 229.5	**<0**.**001**
L2 and 3	3.05 ± 2.95	10.28 ± 1.39	[ + 237.1]	**<0**.**001**

Data reported as mean ± SD; MD, L1, and L2 and 3 are 10-4 mm^2^/s; (R) for right side and (L) for left side. *P*-values in bold are significant.

*Percentage difference for NPH vs. controls. Percentage differences in [] are the largest absolute value/predominant difference used to determine position in the Periodic Table algorithm.

### 3.3 Corpus callosum and periventricular white matter

**FIGURE 1:**
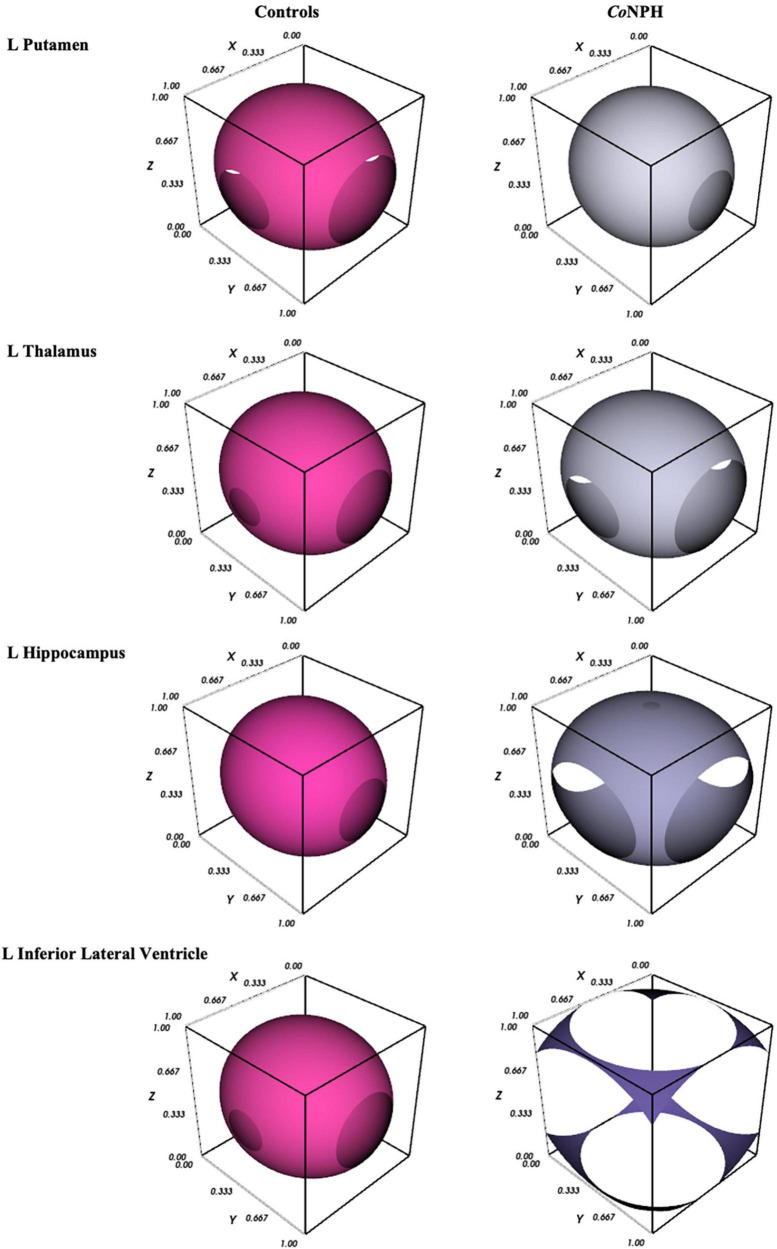
3D graphical representation of white matter integrity in *Co*NPH vs. controls (described in section “2.4 Graphical visualization of DTI profiles”), where the 3D contour plot illustrates the level of structural integrity/tissue distortion.

Fractional anisotropy in the anterior corpus callosum (*p* = 0.011) and posterior corpus callosum (*p* = 0.005) in *Co*NPH was significantly higher than that of controls (Table 4). FA was also higher in *Co*NPH in the central corpus callosum, although it did not reach significance. L1 was significantly higher in *Co*NPH in the central corpus callosum (*p* = 0.002) and posterior corpus callosum (*p* = 0.003). In the lateral ventricles and inferior lateral ventricles, MD, L1, and L2 and 3 were significantly higher in the *Co*NPH group (all *p* < 0.001) while FA was lower (Figure 1). Differences in FA were significant in the lateral ventricles and left inferior lateral ventricle (Table 4).

**TABLE 4:** Diffusion tensor imaging (DTI) metrics for the corpus callosum and periventricular white matter in NPH patients and controls.

Structure	Controls	NPH	% difference*	*p*
**Anterior corpus callosum**				
FA	0.291 ± 0.130	0.390 ± 0.089	[ + 33.8]	**0**.**011**
MD	16.00 ± 6.76	15.17 ± 1.95	−5.23	1.000
L1	20.24 ± 7.63	21.49 ± 2.72	+ 6.16	0.506
L2 and 3	13.89 ± 6.44	12.01 ± 1.86	−13.5	0.518
**Central corpus callosum**				
FA	0.265 ± 0.179	0.375 ± 0.067	[ + 41.8]	0.047
MD	13.77 ± 5.58	15.20 ± 3.12	+ 10.4	0.089
L1	17.18 ± 5.95	21.17 ± 3.25	+ 23.2	**0**.**002**
L2 and 3	12.06 ± 5.73	12.22 ± 3.11	+ 1.29	0.404
**Posterior corpus callosum**				
FA	0.215 ± 0.194	0.358 ± 0.138	[ + 66.8]	**0**.**005**
MD	11.99 ± 3.37	13.52 ± 5.19	+ 12.8	0.119
L1	14.51 ± 3.88	19.14 ± 6.92	+ 32.0	**0**.**003**
L2 and 3	10.73 ± 3.82	10.71 ± 4.50	−0.20	0.719
**Lateral ventricle (R)**				
FA	0.231 ± 0.068	0.164 ± 0.010	−29.1	**0**.**003**
MD	14.35 ± 6.26	27.77 ± 3.72	+ 93.5	**<0**.**001**
L1	17.33 ± 6.78	2.40 ± 4.23	+ 87.0	**<0**.**001**
L2 and 3	12.86 ± 6.01	25.46 ± 3.47	[ + 97.9]	**<0**.**001**
**Lateral ventricle (L)**				
FA	0.225 ± 0.057	0.161 + 0.013	−28.3	**0**.**003**
MD	14.49 ± 6.50	27.66 ± 4.67	+ 90.9	**<0**.**001**
L1	17.48 ± 7.11	32.29 ± 5.47	+ 84.7	**<0**.**001**
L2 and 3	12.99 ± 6.20	25.34 ± 4.28	[ + 95.1]	**<0**.**001**
**Inferior lateral ventricle (R)**				
FA	0.229 ± 0.081	0.195 ± 0.045	−14.9	0.089
MD	10.72 ± 4.84	23.52 ± 4.18	+ 119.3	**<0**.**001**
L1	13.17 ± 5.58	28.03 ± 4.54	+ 112.8	**<0**.**001**
L2 and 3	9.50 ± 4.50	21.26 ± 4.04	[ + 123.8]	**<0**.**001**
**Inferior lateral ventricle (L)**				
FA	0.228 ± 0.071	0.184 ± 0.027	−19.5	**0**.**017**
MD	10.74 ± 4.44	24.12 ± 3.76	+ 124.6	**<0**.**001**
L1	13.20 ± 5.19	28.48 ± 4.13	+ 115.7	**<0**.**001**
L2 and 3	9.51 ± 4.11	21.94 ± 3.59	[ + 130.7]	**<0**.**001**

Data reported as mean ± SD; MD, L1, and L2 and 3 are 10-4 mm^2^/s; (R) for right side and (L) for left side. *P*-values in bold are significant.

*Percentage difference for NPH vs. controls. Percentage differences in [] are the largest absolute value/predominant difference used to determine position in the Periodic Table algorithm.

### 3.4 Subcortical deep gray matter structures

Diffusion tensor imaging metrics for the subcortical deep gray matter structures are shown in Table 5. NPH and control groups differ most significantly in the hippocampus (Figure 1), with significantly lower FA and higher MD, L1, and L2 and 3 in the NPH group (*p* < 0.001 for all). In the caudate bilaterally, FA was significantly lower in NPH than controls, with small differences in MD, L1, and L2 and 3. All measures in the thalamus were higher in *Co*NPH, with L1 being the predominant difference in the right thalamus and FA in the left thalamus. Significant differences in MD and L2 and 3 were noted in the left but not right putamen. In the left and right pallidum, FA and L2 and 3 were significantly higher than in controls (Supplementary Table 1).

**TABLE 5:** Diffusion tensor imaging (DTI) metrics for subcortical deep gray matter structures in NPH patients and controls.

Structure	Controls	NPH	% difference*	*p*
**Caudate (R)**				
FA	0.294 ± 0.077	0.234 ± 0.042	[−20.3]	**0**.**014**
MD	12.77 ± 2.93	13.79 ± 2.29	+ 7.95	0.190
L1	16.19 ± 3.55	16.84 ± 2.40	+ 4.03	0.445
L2 and 3	11.07 ± 2.71	12.26 ± 2.26	+ 10.8	0.153
**Caudate (L)**				
FA	0.295 ± 0.064	0.247 ± 0.047	[−16.2]	**0**.**030**
MD	13.06 ± 2.88	12.78 ± 1.55	−2.09	0.876
L1	16.55 ± 3.55	15.84 ± 1.79	−4.25	0.389
L2 and 3	11.31 ± 2.63	11.25 ± 1.53	−0.50	0.814
**Thalamus (R)**				
FA	0.263 ± 0.076	0.307 ± 0.035	+ 16.6	0.048
MD	10.46 ± 2.22	12.09 ± 1.66	+ 15.7	**0**.**007**
L1	13.02 ± 2.56	15.65 ± 1.84	[ + 20.3]	**<0**.**001**
L2 and 3	9.18 ± 2.18	10.31 ± 1.58	+ 12.4	**0**.**033**
**Thalamus (L)**				
FA	0.241 ± 0.072	0.337 ± 0.053	[ + 40.0]	**<0**.**001**
MD	10.63 ± 2.03	11.72 ± 1.23	+ 10.2	**0**.**012**
L1	13.01 ± 2.05	15.54 ± 1.06	+ 19.4	**<0**.**001**
L2 and 3	9.44 ± 2.13	9.81 ± 1.37	+3.88	0.196
**Putamen (R)**				
FA	0.287 ± 0.098	0.286 ± 0.051	−0.30	0.695
MD	11.08 ± 3.93	9.44 ± 2.72	−14.8	0.127
L1	14.14 ± 4.55	12.14 ± 2.83	−14.1	0.122
L2 and 3	9.56 ± 3.70	8.09 ± 2.67	[−15.3]	0.137
**Putamen (L)**				
FA	0.284 ± 0.080	0.295 ± 0.055	+3.90	0.405
MD	11.59 ± 4.16	9.14 ± 2.30	−21.1	**0**.**028**
L1	14.73 ± 4.91	11.87 ± 2.35	−19.4	0.055
L2 and 3	10.02 ± 3.86	7.78 ± 2.29	[−22.4]	**0**.**024**
**Hippocampus (R)**				
FA	0.237 ± 0.065	0.173 ± 0.061	−26.8	**0**.**002**
MD	9.55 ± 2.47	14.54 ± 1.02	+ 52.3	**<0**.**001**
L1	11.82 ± 3.01	17.08 ± 1.49	+ 44.4	**<0**.**001**
L2 and 3	8.41 ± 2.25	13.27 ± 1.00	[ + 57.9]	**<0**.**001**
**Hippocampus (L)**				
FA	0.233 ± 0.060	0.166 ± 0.057	−28.7	**0**.**001**
MD	9.79 ± 2.02	14.93 ± 1.011	+ 52.6	**<0**.**001**
L1	12.08 ± 2.62	17.42 ± 1.69	+ 44.2	**<0**.**001**
L2 and 3	8.64 ± 1.78	13.69 ± 0.818	[ + 58.5]	**<0**.**001**

Data reported as mean ± SD; MD, L1, and L2 and 3 are 10-4 mm^2^/s; (R) for right side and (L) for left side. *P*-values in bold are significant.

*Percentage difference for NPH vs. controls. Percentage differences in [] are the largest absolute value/predominant difference used to determine position in the Periodic Table algorithm.

## 4 Discussion

We examined cortical and subcortical structural volumes and generated DTI profiles to characterize brain injury in patients with Complex NPH (*Co*NPH) vs. cognitively normal controls from ADNI. Imaging studies in *Co*NPH are rare; we believe this to be the first study showing the concurrent use of both methodologies in this cohort. Patients with *Co*NPH present with symptoms, signs and radiological features like those typical of Classic NPH (*Cl*NPH) but also have the significant clinical burdens from comorbidities such as neurodegenerative disease and vascular risks. They demonstrate far less CSF responsiveness than cohorts of *Cl*NPH; we have previously published results of formal clinical testing via an NPH programme for the patients in this study, supplemented by research imaging and genotypic biomarkers. In this study, we built upon our prior understanding of such testing by documenting the neuroanatomical and microstructural alterations in *Co*NPH. As their DTI interpretation as compared to local controls are known, we then used our findings to validate and expand upon the Periodic Table of DTI Elements (Keong et al., 2022; reproduced here in Tables 6, 7) by comparing them to a set of healthy volunteers from the ADNI open-access dataset. We term this concept “controls-in-common”; by using this strategy, we hope to increase the accessibility of the Periodic Table of DTI Elements to a wider group of community users. Here, we discuss how this methodology situates the *Co*NPH cohort within the spectrum of NPH.

**TABLE 6:** Hierarchical algorithm for mapping DTI profiles to the Periodic Table.

**For differences between patients and controls:**				**Algorithm**	**Position**
A1			No distinct morphological DTI profiles (A2–A6) and no significant changes. There is a presumption of white matter integrity to avoid *over*-interpretation.	⇒	*Order I*
A2			Contradictory differences, small *change in* FA but significant ↓ MD and significant ↓ L1 and/or L2 and 3. *In recovery, ↑ FA, significant ↑ L1 and*↓ *L2 and 3*	⇒	*Order II*
A3			DTI profile of *predominant/significant ↑* L1 or predominant ↓ L2 and 3		
	Bi		Disproportionate ↑ L1 ≥ 2.0-fold vs. MD/L2 and 3 changes	⇒	*Order IV*
	Bii		Predominant ↑ L1 or predominant ↓ L2 and 3, with significant ↑ FA/MD	⇒	*Order V*
	Biii		Predominant ↑ L1 with ↑ L2 and 3, ↑ FA/MD	⇒	*Order V*
A4			DTI profile of *predominant/significant ↑* L2 and 3		
	Bi		Highly disproportionate ↑ L2 and 3 ≥ 2.5-fold vs. MD/L1 changes	⇒	*Order VI*
	Bii		Disproportionate ↑ L2 and 3 ≥ 1.5 to < 2.5-fold vs. MD/L1 changes	⇒	*Order VII*
	Biii		↑ L2 and 3 < 1.5-fold vs. MD/L1 changes, ↑ FA ↑ MD ↑ L1	⇒	*Order VII*
A5			Global DTI profile of worsening = concurrent changes seen of ↓ FA with ↑ MD ↑ L1 ↑ L2 and 3; *But if -*	⇒	*Order VIII*
	Bi		Predominant ↑ L2 and 3, follow algorithm above, *except if*↓ FA or ↑ MD *highest % value, then follow A6 below*	*A4Bi and A4Bii*	
	Bii		↑ L2 and 3 < 1.5-fold, ↓ FA not significant but significant ↑ MD and L1	⇒	*Order VIII*
		Ci	If significant ↓ FA, follow algorithm below	*A6Bii and A6Ci*	
A6			Predominant/significant *individual DTI measure of Global change* (i.e., FA/MD)		
	Bi		Predominant ↑ FA/↓ MD (highest % value) Predominant ↑ FA (highest % value) ↑ FA (or predominant) and ↑ MD (or predominant); (highest % value)	*A3Bii-iii and A4 Bii-iii*	*Follow algorithm for 2nd most predominant (non-FA/MD) measure*
	Bii		↓ FA and predominant ↑ MD (highest % value) Predominant ↓ FA/↓ L1 (highest % value)	⇒ ⇒	*Order VIII* *Order VIII*
A6 cont’		Ci	Significant ↓ FA and		
			a. Global DTI profile *(see A4/A5), disproportionate ↑ L2 and 3* b. Global DTI profile but not matching Order VI/VII c. At-risk profile: ↓ L1 ↑ L2 and 3 d. Significant ↓ MD and L1	⇒ ⇒ ⇒ ⇒	*Order VI/VII* *Order VIII* *Order VIII* *Order X*

Differences between patient cohorts vs. healthy controls at baseline were mapped (Tables 3–5).

For consistency and reproducibility of interpretation, the Periodic Table requires a Hierarchical Algorithm:

0. To solve the Order of the periods:

a. Tables 3–5 for Differences between Patients vs. Controls (for Changes between Cohorts vs. Themselves, refer to Keong et al., 2022).

b. Resolve the Algorithm in order of A) Morphological Profiles, B) Thresholds and C) Significance of Changes.

A. Firstly, describe DTI profiles by their concurrent directions and magnitude of changes.

What morphological descriptor (A2–A6) best describes the DTI profiles seen? If none match, A1 is the default position.

B. Within the morphological descriptor (A2–A6), differentiate DTI profiles by their thresholds for proportions of changes. Disproportion = compare non-FA changes; divide the highest % value by the lowest % value (irrespective of +/– direction).

C. Lastly, resolve the remaining DTI profiles by the level of significance of their changes (Tables 2–4).

D. For conflicts that remain unresolved after steps A–C, describe differences between cohorts by up to two most favourable (lowest ordered) positions.

**TABLE 7:** Recurring common properties of DTI profiles, arranged by expected order of white matter reversibility (for references supporting this interpretation, refer to Keong et al. (2022).

Order	Occurrence	White matter injury patterns
I.	Difference/change	Preserved integrity
II.	Difference/change	Consistent with a range of processes implied by the mechanisms of Neural Repair
III.	Change only	Improvement in compression
IV.	Difference	Compression
V.	Difference	Stretch/compression
VI.	Difference/change	Distortion predominantly due to fluid and/or Post-operative hydrocephalus
VII.	Difference	Edema and/or loss of integrity
VIII.	Difference/change	White matter at-risk of injury disruption due to compression/stretch/edema and/or loss of integrity/atrophy
IX.	Change only	Neuronal degeneration
X.	Difference/change	Swelling/hyper-acute/acute and/or irreversible injury

### 4.1 Interpretation of structural volumetric results

We found that, despite the challenges confounding their assessment, imaging features of this cohort of *Co*NPH demonstrated consistency and agreement with published work on *Cl*NPH. Firstly, we confirmed that patients with *Co*NPH had significant ventriculomegaly, with 180% to 360% differences vs. healthy controls. There were significant differences in patients with *Co*NPH vs. healthy controls for almost all imaging measures in whole brain white matter and cortical matter. We found these structural volumes to be reduced in the patient group. This was true of right- and left-sided measures and across supra- and infra-tentorial spaces in the brain. The corpus callosum, a key white matter tract known to be at risk in both acute and chronic presentations of hydrocephalus, exhibited significantly reduced volumes along its tract. This was consistent from the different sections independently sampled (anterior, middle and posterior corpus callosum). Subcortical deep gray matter (SDGM) structural volumes were also greatly affected. Patients with *Co*NPH showed significantly reduced volumes for all structures except the right thalamus, right putamen and the caudate.

Widespread structural volumetric loss is a feature of neurodegenerative diseases and ageing but reductions in structural volumes are also consistent with shunt responsive NPH. Importantly, it has been shown that shunting has the effect of increasing volumes of SDGM structures, specifically the thalamus and hippocampus (Peterson et al., 2019). Pre-operatively, the mechanism underpinning reductions of volumes in NPH is thought to be reductions in cerebral blood flow (CBF). Reduced CBF in NPH has been well-corroborated using differing imaging techniques such as Dynamic Susceptibility Contrast (DSC) MRI (Ziegelitz et al., 2014), PET (Owler et al., 2004) and Arterial Spin-Labeling (ASL) perfusion MR imaging (Virhammar et al., 2017). Apart from the caudate, findings from our study of reduced volumes in the SDGM structures such as the thalamus and lentiform nucleus, global and periventricular white matter, cortical and cerebellar regions match areas described to have reduced CBF in work by other groups (Owler and Pickard, 2001; Owler et al., 2004; Ziegelitz et al., 2014; Virhammar et al., 2017). By contrast, Agerskov et al. (2020) found no differences in diffusion (apparent diffusion coefficient, ADC) and perfusion (DSC, relative CBF) between patients with iNPH and controls at baseline but improvements in both in the mesencephalon and pons post-operatively, with the rate of rCBF increase correlating to degree of clinical improvement. Using single photon emission computed tomography (SPECT) imaging, Kanemoto et al. (2019) found reduced apathy post-shunting associated with increased regional CBF (rCBF) in the right caudate nucleus. However, vascular risk factors are highly represented in NPH and reductions of CBF may reflect this relationship; improvements in CBF post-operatively may not therefore be directly correlated to improved clinical outcomes. Indeed, Virhammar et al. (2020) found CBF did not increase after shunting in any of the regions above, casting doubt on the utility of ASL in the pre-operative work-up of NPH and perhaps the notion of hypoperfusion as the sole driver of pathophysiological processes triggering the NPH syndrome.

Yet, specific structural patterns of volumetric findings have been described in NPH. Ishii et al. (2008) demonstrated significantly increased gray matter volumes in the medial, lateral parietal and medial frontal lobes and tightness of sulci at the high convexity; the latter supports the diagnosis of NPH according to several criteria (Mori et al., 2012; Kockum et al., 2020; Nakajima et al., 2021). They also found significantly reduced thalamic, caudate head and periventricular gray matter volumes using voxel-based morphometry (VBM). Using VBM, Lv et al. (2022) recently confirmed the presence of both patterns of structural volumetric changes, with decreased gray matter volumes of iNPH in bilateral temporal lobes, bilateral hippocampi, bilateral thalami, bilateral insulae, left amygdala, right lenticular nucleus, right putamen and cerebella and increased gray matter volumes in the bilateral paracentral lobules, precuneus, bilateral supplementary motor areas, medial side of the left cerebral hemisphere, median cingulate and paracingulate gyri. It was also possible using meticulous neuropsychological testing and FSL-based segmentation to show volumetric data could be correlated to neuropsychological scores (Peterson et al., 2019). In summary, greater volumes correlated with improved performance on cognitive testing and reduced apathy. Pre-shunting nucleus accumbens volumes showed significantly positive correlations to verbal learning and memory; post-shunting caudate volumes were significantly positively correlated with global cognitive functioning [Mini-Mental State Examination (MMSE) scores] and semantic fluency.

Our findings of widespread and significant reductions in SDGM structures match such established literature and support our working hypotheses that even clinically challenging cohorts such as *Co*NPH still retain imaging features associated with *Cl*NPH. The asymmetrical reductions in volumes of the thalami and putamina (significant differences only in left-sided structures) may reflect differing thresholds of injury, rather than a predilection for pathophysiological processes in NPH to affect left-sided structures. Indeed, both right-sided structures demonstrated the same directional differences, albeit non-significantly. However, this is not the case for the caudate, which instead showed no directional trends. Our results of non-significant differences in caudate volumes are surprising given that changes in basal ganglia structural volumes have been rather consistently reported in NPH studies (DeVito et al., 2007; Ishii et al., 2008; Peterson et al., 2019; Lv et al., 2022) and increases in caudate rCBF were correlated only with patients who showed improvements in apathy post-operatively (Kanemoto et al., 2019). We suggest two alternative hypotheses for this finding. The first would be that changes in the caudate may be the most sensitive at distinguishing between cohorts of *Cl*NPH and *Co*NPH. Significant reductions in the caudate that improve post-surgical intervention may demonstrate evidence for the concept of “reversible brain injury” if conversely, the absence of this finding suggests the predominant presence of “irreversible brain injury.” One way to confirm this would be to compare caudate volumes for both cohorts of *Cl*NPH and *Co*NPH separately against a common cohort of controls. We intend to perform such work as a follow-on from this study to examine this possibility.

The second hypothesis we wish to offer is that the results of caudate volumes may be impacted upon by contradictory findings when multiple pathophysiological processes occur concurrently at the microstructural level. As both increases and decreases in gray matter volumes have been reported by other groups (Ziegelitz et al., 2014; Lv et al., 2022), such changes occurring concurrently in the caudate or any other SDGM structure may influence their volumetric results. Findings presented in the Peterson et al., study provide support for this hypothesis. In that cohort, there were significant reductions in all SDGM structures except the amygdala. By contrast, contradictory changes were noted in the pallidum; greater post-shunting volumes were associated with poorer performance on the MMSE and increased self-rated apathy. Yet, the amygdala and pallidum have not been known to be specifically spared by NPH patterns of injury; Lv et al. (2022) found significant reductions in amygdala volumes, whilst significant reductions in the CBF of the lentiform nucleus were reported by both Ziegelitz et al. (2014) and Virhammar et al. (2017).

We believe the latter hypothesis to be more immediately plausible as contradictory findings from concurrently occurring pathological processes have been shown in NPH. Using DTI, we found evidence that the caudate was not spared but rather, demonstrated alterations consistent with edema and loss of microstructural integrity.

### 4.2 Expanding the periodic table of DTI elements and its application in CoNPH

We found evidence for widespread microstructural alterations in patients with *Co*NPH compared to healthy controls. In summary, there were significant differences in DTI profiles in all whole white matter and cortical matter regions in the cerebral hemispheres and bilateral cerebella (Table 8). The corpus callosum demonstrated two differing but significant patterns of injury on DTI profiles (Table 9); both were successfully resolved via one algorithmic solution (A6Bi to A3Bii) to the same Order, using the Periodic Table of DTI Elements. By contrast, periventricular white matter demonstrated two differing and significant DTI profiles but these patterns required two algorithmic solutions (A5Ci to A6Cib and A5Bii) to resolve them, also to the same Order (Table 9). Additionally, the adjacent Orders seen in the right vs. left amygdala confirm that DTI profiles in each side can be resolved independently. The SDGM structures showed significantly different DTI profiles in patients with *Co*NPH compared to healthy controls, apart from the right putamen and bilateral nuclei accumbens. Using the Periodic Table of DTI Elements, we found the reasons behind this to be strikingly different. The nuclei accumbens showed microstructural evidence of irreversible brain injury whereas the bilateral putamina were revealed to the SDGM structures most preserved in *Co*NPH (Table 10).

**TABLE 8:** Diffusion tensor imaging (DTI) profile differences for NPH vs. controls in white matter and cortical matter in the supratentorial vs. infratentorial spaces mapped to the Periodic Table.

Structure	Solution to resolve algorithm	Position by Order of the Periods
Cerebral white matter (R)	A6Bi and A3Bii/Biii	*Order V*
Cerebral white matter (L)	A6Bi and A3Bii/Biii	*Order V*
Cerebral cortex (R)	A4Biii	*Order VII*
Cerebral cortex (L)	A4Biii	*Order VII*
Cerebellum white matter (R)	A6Bi and A3Bii/Biii	*Order V*
Cerebellum white matter (L)	A6Bi and A3Bii/Biii	*Order V*
Cerebellum cortex (R)	A4Biii	*Order VII*
Cerebellum cortex (L)	A4Biii	*Order VII*

**TABLE 9:** Diffusion tensor imaging (DTI) profile differences for NPH vs. controls in the corpus callosum and periventricular white matter mapped to the Periodic Table.

Structure	Solution to resolve algorithm	Position by Order of the Periods
Anterior corpus callosum	A6Bi and A3Bii	*Order V*
Central corpus callosum	A6Bi and A3Biii	*Order V*
Posterior corpus callosum	A6Bi and A3Bii	*Order V*
Lateral ventricle (R)	A5Ci and A6Cib	*Order VIII*
Lateral ventricle (L)	A5Ci and A6Cib	*Order VIII*
Inferior lateral ventricle (R)	A5Bii	*Order VIII*
Inferior lateral ventricle (L)	A5Ci and A6Cib	*Order VIII*

**TABLE 10:** Diffusion tensor imaging (DTI) profile differences for NPH vs. controls in subcortical deep gray matter structures mapped to the Periodic Table.

Structure	Solution to resolve algorithm	Position by Order of the Periods
Caudate (R)	A5Ci and A6Bii	*Order VIII*
Caudate (L)	A5Ci and A6Bii	*Order VIII*
Thalamus (R)	A3Bii/Biii	*Order V*
Thalamus (L)	A6Bi and A3Bii/Biii	*Order V*
Putamen (R)	A1	*Order I*
Putamen (L)	A2	*Order II*
Pallidum (R)	A3Bii	*Order V*
Pallidum (L)	A6Bi and A3Bii	*Order V*
Hippocampus (R)	A5Ci and A6Cib	*Order VIII*
Hippocampus (L)	A5Ci and A6Cib	*Order VIII*
Amygdala (R)	A4Biii	*Order VII*
Amygdala (L)	A4Bii/A5	*Order VII/VIII*
Accumbens (R)	A6Bii	*Order VIII*
Accumbens (L)	A6Bii	*Order VIII*

Our work on DTI profiles has been published elsewhere (Keong et al., 2017, 2022; Lock et al., 2019). In brief, DTI profiles are a methodology of describing concurrent changes occurring across different DTI measures and distilling such complexity into their predominant direction and magnitude of changes for consistency of interpretation. We have previously reported that DTI profiles of *Cl*NPH showed at least three patterns of white matter injury co-existing. Here, we found that DTI profiles of *Co*NPH showed features of both reversible and irreversible patterns of injury. There was an overlap between DTI profiles in *Co*NPH and DTI profiles of both cohorts of *Cl*NPH and AD, as previously published by our group (Keong et al., 2022).

We believe the utility of DTI in NPH to be that it allows for the exploration of multiple theories of NPH pathogenesis that may be equally plausible as the main drivers provoking the changes seen in literature and in this study (Keong et al., 2016; Grazzini et al., 2020). Tissue distortion, caused by progressive ventriculomegaly, may affect the course of penetrating arteries supplying the SDGM structures and periventricular white matter. White matter blood flow is known to be reduced in NPH, altered in a U-shaped distribution from the lateral ventricles to subcortex (Momjian et al., 2004). The effect of stretch/compression leads to disruption of the arterial supply and reduction of CBF in a manner that could be reversible with surgery. Another theory of NPH pathogenesis postulates that transependymal leakage of CSF results in reversal of interstitial fluid flow, leading to edema in the local tissues and periventricular white matter. The effect of this accumulation of fluid may manifest in several ways, i.e., as a cause of—(i) periventricular edema, (ii) compression of penetrating arteries and/or (iii) inefficient retention or failure of drainage of vasoactive metabolites. Both tissue distortion and interstitial edema may disrupt the white matter connections serving the subcortical structures and cortex in ways consistent with NPH injury patterns reported by our group and others (Hattori et al., 2012; Keong et al., 2017).

The challenges of utilizing DTI to study the spectrum of NPH encompass several technical factors, biological considerations and scientific/clinical concerns. They can be summarized as the following conceptual list of problems: (Keong et al., 2016; Kok et al., 2022)–(i) “the problem of the scanner”—DTI measures are dependent on machine-specific/technical specifications for scanning acquisition and the effect of such variations between sites may be hard to quantify/correct for, (ii) “the problem of gold standard”—DTI output is dependent upon processing software techniques for which there are varying advantages and disadvantages but no single, unifying standard, (iii) “the problem of DTI methodology”—DTI metrics are subject to biological confounders such as multiple pathophysiological processes or crossing fibers occurring within the sample/region-of-interest, (iv) “the problem of cohorts”—DTI results can be inconsistent both within and across patient groups within the same disease process, as well as over time, (v) “the problem of consistency of interpretation”—DTI results can appear contradictory across the full panel of DTI measures even within known functional neuroanatomical groupings, (vi) “the problem of lack of samples”—there are insufficient published DTI samples of clinical cohorts who can represent distinct milestones within the spectrum of reversible to irreversible injury and (vii) “the problem of lack of comparators”—unlike structural imaging measures, individual DTI metrics are not clinically comparable across sites, leading to a lack of baseline reference values to be used in common.

### 4.3 The Periodic Table of DTI Elements supports the concept of a translational taxonomy

As a solution to address the list of DTI problems beyond the scanner and pre-/post-processing techniques, we proposed a novel taxonomic framework termed the Periodic Table of DTI Elements (Keong et al., 2022). By resolving commonly recurring patterns of DTI morphological changes as “Periods” and by further arranging them into “Orders,” we intend to provide for a more transparent and reproducible methodology for DTI interpretation. Crucially, the Orders are arranged sequentially in a spectrum of reversible to irreversible patterns of injury. As these morphological descriptors are declared *a priori*, we believe it is possible to situate patient cohorts within their clinically relevant contexts for more precise interpretations of DTI findings. In our previous work (Keong et al., 2022), we considered familial neuroanatomical ROIs, comprising a model of white matter tracts at risk in hydrocephalus. To render the conceptual framework of the Periodic Table of DTI Elements more relevant to *Co*NPH, where substantial overlay with conditions including neurodegenerative disorders and vascular risks exists, it was necessary to expand upon our work to include subcortical gray matter and cortical structures.

In this iteration, we included a further descriptor of a morphological DTI profile heretofore unseen in our previous work. However, we situated this new profile, that of concurrent increases in FA, MD, L1 and L2 and 3, within the classification scheme already proposed. We did so by refining the previously defined morphological DTI profiles and providing clarifications for the contexts in which they occur. In order of reversibility to irreversibility of injury, the following DTI descriptors were declared:–(i) A1; no distinct morphological DTI profiles (A2–A6) and no significant changes, (ii) A2; contradictory differences, small change in FA, significant reduction in MD and signification reduction in L1 and/or L2 and 3, (iii) A3; DTI profile driven by predominant/significant increase in L1 and/or predominant reduction in L2 and 3, (iv) A4; DTI profile driven by predominant/significant increase in L2 and 3, (v) A5; Global DTI profile of worsening, i.e., a concurrent decrease in FA with increases in MD, L1 and L2 and 3 and (vi) A6; predominant/significant individual DTI measure of global change, (i.e., FA/MD).

By applying the strategy of this Periodic Table of DTI Elements, we made the following observations in our study. Although both whole brain white matter and cortical matter in the cerebral hemispheres and bilateral cerebella demonstrated significantly differing DTI profiles in *Co*NPH vs. controls, there were two distinct periodic patterns of injury. Cerebral and cerebellar white matter demonstrated more potential reversibility of injury (Order V, overlap with *Cl*NPH cohort) as compared to cerebral and cerebellar cortices (Order VII, overlap with AD cohort; Keong et al., 2022). This may be influenced by the injury of the white matter connections subserving these areas; the “connectivity end organs” being more affected than white matter tracts, which may have more capacity for recovery or resilience. This likely explains the finding of structural volumetric and CBF reductions in the cerebella at baseline (Owler et al., 2004; Virhammar et al., 2017; Lv et al., 2022), as well as ADC and rCBF improvements in the mesencephalon and pons post-operatively in shunt responders (Agerskov et al., 2020). Although relatively remote to the areas of greatest tissue distortion due to the risk from progressive ventriculomegaly (adjacent to the lateral ventricles), these regions may still be affected due to receiving projections from the motor cortex and corticospinal tract and may be influenced by the impact of white matter injury and/or recovery. However, when white matter tracts at-risk were considered as individual ROIs, they were also affected to differing degrees. The corpus callosum ROIs were more preserved (Order V) than the periventricular white matter ROIs (Order VIII; see Table 8). We believe these findings are consistent with the theories of NPH pathogenesis as described above.

Interestingly, the corpus callosal findings in *Co*NPH fall within the range of Orders we previously reported for a cohort of *Cl*NPH (Keong et al., 2022), albeit as an incomplete match to them. In this study, we did not find evidence of the morphological DTI profile of Order VI (A4Bi). This profile, of highly disproportionate increased L2 and 3 changes appeared to be consistent across multiple familial ROIs and was present pre- and post-shunting in our cohort of *Cl*NPH. We had suggested this to be a classic finding of DTI profiles in the context of patients with hydrocephalus. Its absence in our current study cohort may be interpreted in two ways. Firstly, it is possible that this morphological DTI feature may confer a more “reversible brain injury” pattern; this may be lacking in the *Co*NPH cohort, who are far less responsive to CSF interventions than the cohort with *Cl*NPH. If so, we might expect to see this Order represented in some of the individual patients (the minority) of the *Co*NPH who were CSF-responsive. Secondly, in this iteration of the Periodic Table of DTI Elements, to expand its relevance to a larger community of users, it was necessary to use an open-access dataset. As discussed above, DTI metrics may not be portable from site to site and the results of this comparator group may have affected the proportionality required for the Periodic Table concept to work. We hope to examine these possibilities in a cohort of *Co*NPH with larger numbers of patients and by re-comparing *Cl*NPH to the open access controls to provide baseline reference values.

In addition to external consistency to the other published cohorts, we found that describing periodic patterns of injury allowed us to be more internally consistent in our interpretation of DTI results. For example, the corpus callosal ROIs demonstrated differing morphological DTI profiles (anterior corpus callosum vs. central and posterior corpus callosum). These were duly independently resolved in two different ways using the algorithm for the Periodic Table of DTI Elements and found to map to the same Order. Similarly, when the morphological DTI profiles were generated for the SDGM structures, we found that it was possible to both (i) demonstrate right- and left- consistency in Orders derived independently from DTI profiles for each side, as well as (ii) demonstrate the spectrum of reversible to irreversible periodic patterns of injury co-existing within a single cohort of disease. Striving for consistency of reporting DTI findings in this way also assists in situations where there is a possibility of over- or under-interpreting borderline significant results. In this study, we applied a correction with the Benjamini-Hochberg false discovery rate to avoid such concerns; significance was therefore determined at the level of *p* ≤ 0.033. The effect of applying this higher threshold altered the results of two ROIs (central corpus callosum; FA and right thalamus; FA) from significant to non-significant. However, the change in significance for individual measures had no impact on the interpretation of these ROIs via the Periodic Table of DTI Elements. It was possible for DTI findings in each ROI to be resolved via the same steps using the algorithm (central corpus callosum; A6Bi and A3Biii, Order V, and right thalamus; A3Bii/Biii, Order V). As the methodology of the Periodic Table does not rely solely upon the significance (or not) of individual measures, but rather, also classifies DTI findings by their over-arching morphological descriptors, the mapping of all three ROIs remained unchanged. In summary, the Order of the ROIs, according to taxonomy set by the Periodic Table, remained resilient to changes in the threshold level at which findings were deemed significant.

Accordingly, we found that in this cohort of patients with *Co*NPH, the bilateral hippocampi and nuclei accumbens were the most consistently affected by irreversible patterns of brain injury (Order VIII). Hippocampal atrophy is a known hallmark of AD; both pre- and post-shunt nuclei accumbens structural volumes are correlated to performance on cognitive testing in NPH (Peterson et al., 2019) This is consistent for the definition of *Co*NPH we have previously proposed, which situates this cohort at the intersection between both diseases. By contrast, despite significant reductions in left-sided structural volumes, the putamina were the most preserved (Order I/II, respectively). As postulated above, the asymmetric reductions in structural volumes may reflect thresholds of changes due to level of injury. We suggest that this finding is also consistent with theories of NPH pathogenesis; the putamina are the most laterally placed of the SDGM structures and may be least at risk from its tissue distortion caused by progressive ventriculomegaly. The Periodic Table was also useful to confirm the situation in the caudate. It is possible, with the presence of Order VIII, indicating white matter at-risk of injury, that disruption can be due to differing pathophysiological processes, ranging the spectrum from compression/stretch/edema and/or loss of integrity/atrophy. Multiple changes occurring concurrently may alter the caudate structural volumes in opposing directions, effectively canceling out any significant reductions in structural volumes due to reduced CBF. Further work is necessary to confirm the findings above and provide other exceptions.

To summarize, in this study we have found that imaging features of *Co*NPH may be interrogated with structural volumetric and diffusion measures and share similar findings with *Cl*NPH. The utility of DTI is its capacity to contribute to examining for contrasting microstructural changes hypothesized by the known theories of NPH pathogenesis. We suggest also that multiple concurring pathophysiological processes co-exist within the NPH spectrum. Therefore, the separate use of single imaging modalities may be insufficient to describe the breadth of changes occurring in NPH. Here, we have demonstrated the complementary use of volumetric segmentation and DTI to infer changes in both structural neuroanatomy and tissue microstructure. However, it may equally be reasonable to pair other complementary imaging modalities to supplement gaps in and strengthen findings of the methodologies presented in this study. For this to occur, it is necessary to provide transparent frameworks to guide the interpretation of the results of each modality in ways that would be both externally and internally consistent. We believe that by proposing the Periodic Table of DTI Elements, we are contributing toward this ethos. The difference in our approach is that the premise for this taxonomy was of a re-imagining of brain microstructures as a “novel biomaterial,” whose properties, both diffusivity and neural, must first be described and organized into a spectrum of reversible to irreversible injury, before it can be rebuilt into structural architectural “models” of human neurological disease. In developing this premise, we drew inspiration from the first human attempts to organize natural occurring material elements into a common taxonomic framework for use and applied this to our “brain biomaterial”; we term this approach a concept of a “translational taxonomy.” This idea of describing brain tissue as a “material” also has its basis in work by Peña et al. (1999) and others, who have successfully demonstrated the clinical relevance of modeling brain tissue as a poroelastic sponge-like material. The next step in the evolution of the concept of the Periodic Table of DTI Elements may be to pursue further models in materials and natural sciences that may offer meaningful and relevant insights toward the study of human brain injury.

### 4.4 Limitations

Our study is subject to several shortcomings. The *Co*NPH cohort is small, which reflects the low numbers of patients presenting with this subtype within the NPH spectrum. However, the advantage of this cohort is that we have previously published our clinical, radiological and genotypic biomarkers characterizing their specific risks. We have also reported their DTI profiles compared to local healthy controls. In this study, we wished to expand upon the potential accessibility of the Periodic Table of DTI Elements by using an open-access dataset for healthy control subjects. As individual DTI metrics may not be clinically comparable across scanning sites, it was thus critical that CSF responsiveness and DTI profiles for this patient cohort be documented *a priori*. Further work toward the Periodic Table of DTI Elements needs to be undertaken to re-compare our published *Cl*NPH cohort against this new baseline of healthy control subjects before expanding its use to further clinical groups. Following this, another consideration would be to examine if the Orders of the Periodic Table directly correlate in a meaningful way to minimal clinically important differences in the spectrum of reversible to irreversible brain injury. Currently, we have access to limited datasets to test such hypotheses; NPH cohorts are small and automated volumetric segmentation methods fail in severe ventriculomegaly. Therefore, the aim of expanding the accessibility of the methodology of the Periodic Table is to encourage further collaboration toward a more transparent and reproducible framework for the utility of DTI in the study of brain injury. Our intention would be that this model be regarded as a prototype that could be refined and improved with the input and contribution of a wider community of users to further address its current shortcomings.

## 5 Conclusion

We previously proposed a novel taxonomic framework to describe patterns of DTI profiles across a spectrum of acute-to-chronic brain injury. We termed this strategy a Periodic Table of DTI Elements, the first attempt to organize the interpretation of neural tract patterns by their diffusivity and neural properties. However, the limitation of this approach was twofold; firstly, that it mainly described profiles of white matter injury and secondly, that it required the comparison to local controls and was not generalizable across global communities. Our goal in this study was to expand the utility of this methodology by both (i) interrogating other subcortical and cortical structures of interest and (ii) comparing them to brain metrics in controls derived from an open-access dataset, i.e., promoting the concept of utilizing “controls-in-common.” In this study, we have shown that it is possible to do both. In addition, we have also demonstrated that patients with *Co*NPH share imaging biomarker features that overlap with cohorts of *Cl*NPH and AD. Such understanding is an important step toward characterizing the milestones that describe the continuum of patterns from reversible to irreversible brain injury. Ultimately, our aim is to provide a prototype that could be refined and improved for an approach toward the concept of a “translational taxonomy.”

## Data availability statement

The raw data supporting the conclusions of this article will be made available by the authors upon reasonable request.

## Ethics statement

The studies involving humans were approved by the SingHealth Centralised Institutional Review Board. The studies were conducted in accordance with the local legislation and institutional requirements. The participants provided their written informed consent to participate in this study.

## Author contributions

NK conceptualized the experimental design. CL and NK analyzed the data and wrote and revised the manuscript. ET and NK conceptualized the 3-Dimensional illustrations for the DTI profiles and ET produced the final graphical representations. All authors contributed to the article and approved the submitted version.

## Footnotes

1
adni.loni.usc.edu
