# Supplementary material for: Structural volumetric and Periodic Table DTI patterns in Complex Normal Pressure Hydrocephalus—Toward the principles of a translational taxonomy
Source: Front Hum Neurosci. 2024 Mar 13;18:1188533. doi: 10.3389/fnhum.2024.1188533 (PMC10965785; doi:10.3389/fnhum.2024.1188533)
Supplement: Supplementary file 1 [file Table_1.DOCX]

Supplementary Material

Structural Volumetric and Periodic Table DTI Patterns in Complex Normal Pressure Hydrocephalus – Towards the Principles of a Translational Taxonomy

Christine Lock, Nicole C Keong*

*** Correspondence:** Corresponding Author: nchkeong@cantab.net

# Supplementary Table

**Supplementary Table 1.** DTI values for subcortical deep grey matter structures (pallidum, amygdala, and accumbens) in NPH patients and controls.

| **Structure** | **Controls** | **NPH** | **% difference*** | ***p*** |
| --- | --- | --- | --- | --- |
| Pallidum (R) | |  |  |  |
| FA | 0.291 ± 0.090 | 0.390 ± 0.063 | +34.3 | **0.001** |
| MD | 11.93 ± 5.11 | 8.28 ± 1.08 | -30.6 | **0.007** |
| L1 | 15.17 ± 5.81 | 11.88 ± 1.10 | -21.7 | 0.309 |
| L2and3 | 10.31 ± 4.84 | 6.48 ± 1.17 | [-37.2] | **0.001** |
| Volume | 2005 ± 299 | 1709 ± 361 | -14.8 | **0.024** |
| Pallidum (L) | |  |  |  |
| FA | 0.284 ± 0.095 | 0.371 ± 0.097 | [+30.7] | **0.007** |
| MD | 11.96 ± 5.19 | 9.01 ± 1.88 | -24.7 | 0.100 |
| L1 | 15.14 ± 6.07 | 12.63 ± 1.71 | -16.55 | 0.938 |
| L2and3 | 10.37 ± 4.84 | 7.20 ± 2.08 | -30.6 | **0.014** |
| Volume | 1957 ± 224 | 1525 ± 304 | -22.1 | **<0.001** |
| Amygdala (R) | |  |  |  |
| FA | 0.237 ± 0.097 | 0.237 ± 0.053 | +0.28 | 0.710 |
| MD | 9.70 ± 4.06 | 10.70 ± 1.60 | +10.3 | 0.055 |
| L1 | 12.06 ± 4.90 | 13.23 ± 1.91 | +9.73 | **0.026** |
| L2and3 | 8.53 ± 3.71 | 9.44 ± 1.51 | [+10.7] | 0.117 |
| Volume | 1633 ± 230 | 1323 ± 257 | -19.0 | **<0.001** |
| Amygdala (L) |  |  |  |  |
| FA | 0.233 ± 0.088 | 0.215 ± 0.043 | -7.67 | 0.784 |
| MD | 10.37 ± 4.59 | 11.50 ± 3.93 | +10.8 | 0.081 |
| L1 | 12.78 ± 5.48 | 13.85 ± 4.10 | +8.36 | 0.058 |
| L2and3 | 9.17 ± 4.20 | 10.32 ± 3.85 | [+12.5] | 0.117 |
| Volume | 1445 ± 207 | 1087 ± 208 | -24.8 | **<0.001** |
| Accumbens (R) |  |  |  |  |
| FA | 0.271 ± 0.129 | 0.223 ± 0.041 | [-17.6] | 0.368 |
| MD | 13.40 ± 6.96 | 11.65 ± 6.12 | -13.1 | 0.667 |
| L1 | 16.69 ± 7.80 | 14.00 ± 6.72 | -16.1 | 0.218 |
| L2and3 | 11.76 ± 6.64 | 10.48 ± 5.82 | -10.9 | 0.891 |
| Volume | 442 ± 86.2 | 334 ± 122 | -24.5 | **0.002** |
| Accumbens (L) | |  |  |  |
| FA | 0.294 ± 0.134 | 0.256 ± 0.036 | -12.9 | 0.570 |
| MD | 14.21 ± 7.04 | 11.25 ± 5.67 | -20.8 | 0.240 |
| L1 | 17.86 ± 7.52 | 13.81 ± 6.51 | [-22.7] | 0.050 |
| L2and3 | 12.39 ± 6.91 | 9.98 ± 5.25 | -19.5 | 0.358 |
| Volume | 410 ±109 | 349 ± 67.9 | -14.8 | 0.053 |

Data reported as mean ± SD; MD, L1, and L2and3 are 10^−4^ mm^2^/s; (R) for right side and (L) for left side. P-values in bold are significant. *Percentage difference for NPH vs controls. Percentage differences in [ ] are the largest absolute value/ predominant difference used to determine position in the periodic table algorithm.
